# Forest Walk Methods for Localizing Body Joints from Single Depth Image

**DOI:** 10.1371/journal.pone.0138328

**Published:** 2015-09-24

**Authors:** Ho Yub Jung, Soochahn Lee, Yong Seok Heo, Il Dong Yun

**Affiliations:** 1 Division of Computer and Electronic Systems Engineering, Hankuk University of Foreign Studies, Yongin, Republic of Korea; 2 Department of Electronic Engineering, Soonchunhyang University, Asan, Republic of Korea; 3 Department of Electrical and Computer Engineering, Ajou University, Suwon, Republic of Korea; University of North Carolina, UNITED STATES

## Abstract

We present multiple random forest methods for human pose estimation from single depth images that can operate in very high frame rate. We introduce four algorithms: random forest walk, greedy forest walk, random forest jumps, and greedy forest jumps. The proposed approaches can accurately infer the 3D positions of body joints without additional information such as temporal prior. A regression forest is trained to estimate the probability distribution to the direction or offset toward the particular joint, relative to the adjacent position. During pose estimation, the new position is chosen from a set of representative directions or offsets. The distribution for next position is found from traversing the regression tree from new position. The continual position sampling through 3D space will eventually produce an expectation of sample positions, which we estimate as the joint position. The experiments show that the accuracy is higher than current state-of-the-art pose estimation methods with additional advantage in computation time.

## Introduction

Marker-less human pose estimation has been a highly sought-after goal of computer vision for many years. Automatic and accurate pose estimation provides various application opportunities in video games, security, teleconferences, and automatic photo editing. For these applications, while a pose estimation from a single color image might be the most practical, an accurate method has yet to be developed, especially one that can operate in real time, despite recent advancements.

Meanwhile, the introduction of accurate depth cameras has made real-time human pose estimation a much more feasible problem. Numerous super-real-time methods are introduced [1–7]. Also, a variety of interesting applications became possible with real time pose estimation [8]. Despite their efficiency, there is still demand for further improvement. For example, the recent method of Shotton et al. [5] relies on parallel computation using either a GPU or a multi-core CPU to achieve real-time performance. These resources are not easily available in current embedded platform computing devices such as smart-phones, TVs, or tablets. Reducing computation is especially crucial for mobile devices, since battery life depends on the amount of computation. Also, more importantly, a pose estimation algorithm has to operate simultaneously with multiple applications and games which require a larger portion of computational resources [9].

Many previous body joint localization methods relied on independent per-pixel operations such as body part classification or joint offset regression [5, 6, 10, 11]. By exploiting the parallel computability, the random forests [12] are traversed using GPU or multi-core processors. The random forest leaves are aggregated to find the expectation or the mode, which is designated as the estimated body part joint. However, with pixel-wise random forest, excessive computational resources are committed to larger body parts. Although a uniform sub-sampling is a possible solution for computation time reduction, the uneven allocation of computational cost is still unavoidable.

**Fig 1:**
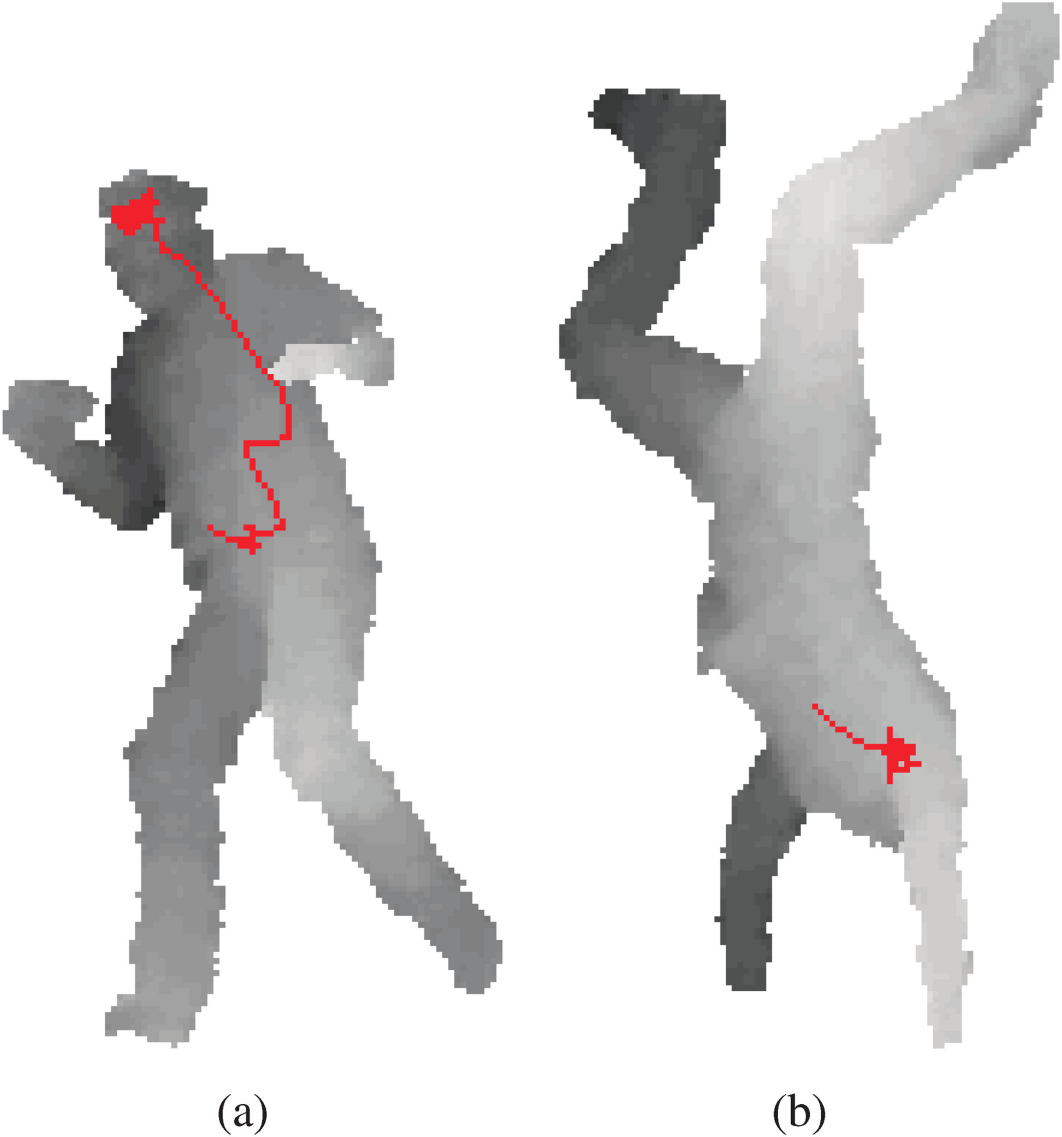
The red lines represent the random forest walks trained to find the position of the head. The random walk starts from the body center in (a). In (b), the head position is found with fewer steps by starting from the chest, which is much closer than the body center.

In this paper, we present a simple, yet powerful discriminative method for body part localization from a single depth image, called the Random Tree Walks (RTW) method [13]. The proposed RTW combines random trees and random walk to train the relative directional path to a specific joint from any point. The RTW comprises two stages. The first is to train a random tree (RT) to estimate the relative direction to the joint from a given point. The second is the joint localization stage, where an initial starting point is moved towards the joint position by random walk in the direction estimated from the trained random regression tree. In each step of random tree walk, we are sampling closer to the targeted body joint. Regardless of the size of body part, the number of random walk can be kept minimum and consistent through all body joints. Fig 1 shows an example of the proposed random tree walk process to estimate position of the head. We can see the path of the walk as the regression tree guides the direction of each step at each point. Furthermore, the kinematic tree of joints can be leveraged by performing RTW sequentially, and by initializing the subsequent RTW’s starting point as the estimated position of a preceding joint. We construct an optimal sequence of joints based on the kinematic tree. The comparison between Fig 1a and 1b demonstrates the efficiency of our method in finding the head position.

Also, we construct and evaluate variants of the proposed method in three different aspects. First, we expand the single tree into a forest. In this case the RTW becomes the Random Forest Walk (RFW). We demonstrate the gain in accuracy in using multiple trees and the trade-off in computation time. Second, we define alternative regression outcomes from each random tree. In one instance, the regression output is randomly selected among multiple candidates constructed from the distribution of training data directions for each leaf node of a RT [13]. In another, a single deterministic output is trained for each RT leaf node. Here, we refer to the former as a *random* tree, while we term the latter as a *greedy* tree. Third, we train the positional offsets to the joints instead of relative directions. By training offsets, a single jump to the correct joint position is possible, but a large jump to an incorrect joint position is also possible.

Unlike the previous methods [5, 6, 10], the trained RT or random forest (RF) is traversed only a fixed number of times for each body joint. Thus, the number of tree traversal is independent of the size of the body parts. The fewer number of RT evaluations result in a large computational gain compared to the previous pixel-wise methods [11]. When optimized in terms of efficiency, the proposed method can operate over 1000 frames per second (fps) on a single core 3.20 GHz CPU with no additional use of GPU or SIMD operations. Considering the omission of parallel computing, the proposed method requires less than 1% of the computation of previous methods, while having equal or comparable overall accuracy. When optimized in terms of accuracy, the proposed method outperforms the state-of-the-art methods.

In summary, we introduce and evaluate four different algorithms for fast 3D pose estimation from a single depth image.

- Random Forest Walk (RFW) is an extension of the random tree walk (RTW) algorithm described in our conference paper [13]. We use random forest instead of random tree to train and sample relative direction to the target joint.
- Greedy Forest Walk (GFW) approach does not select the next direction to the step randomly from distribution as in RFW. The random forest is trained to output a single direction to the target joint in this approach.
- Random Forest Jumps (RFJ) approach trains the offset to the target joint instead of the direction. The training objective is more similar to the previous method in [10]. RFJ can find the correct target joint position in a single jump, but at the same time it can jump to wrong position.
- Greedy Forest Jumps (GFJ) approach is the same as RFJ algorithm except the jumps are not chosen randomly from a probability distribution. Just like GFW, a single offset value is stored in the leaf of regression tree. This method for localization was previously introduced for estimating land-mark position in MR brain image registration [14]. We show that this method is efficient also for localizing body parts during pose estimation.

## Related Works

There are a significant number of papers on human pose estimation problem. We briefly review relevant pose estimation methods from color and depth images, and also examine methodologies related to the proposed pose estimation algorithms.

### Pose Estimation from Color Images

More comprehensive surveys of human pose and action estimation approaches are found in [15, 16]. The problem domains are often divided into pose estimation from a single image, video sequence or multi-view camera. Recent advancements in pose estimation from a single image include poselets [17], the mixture-of-parts model [18] and DeepPose [19]. Pose estimation approaches from video sequences [20–22] employ additional temporal information in the form of background subtraction or body part segmentations. In [20], the body segmented image was used as features, and hashing technique was used to find examples with similar features and poses. In the work of [21], it was shown that the human silhouette is indeed an efficient descriptor for 3D pose recovery. By increasing the view points, more accurate human extraction was possible in works such as that of [23] and [24]. In works including [25] and [26], color invariant depth maps were constructed using efficient stereo matching, which were used to estimate the pose.

In many of the previous works, some form of kinematic constraint is utilized when completing the skeletal frames. In the method of [27], the deformation between body joints was modeled with springs. In the method of [28], non-parametric belief propagation was used over the template detectors to infer skeletal frames. As with many of the above papers, our method redecorate the skeleton based kinematic constraints into sequential body joint estimation.

#### Pose Tracking in Depth Sequence

Compared to color images, depth images provide much more invariant information for pose estimation. In many of the previous works, a pose is estimated by minimizing the distance between a human model and the depth map [2, 3, 29–32]. The minimization functions found in generative models are often difficult to be optimized due to many local minima. The energy minimization scheme usually relies on a good initialization state which can be obtained from the previous frame’s pose. Therefore, these methods, based on generative models for pose estimation, are essentially tracking methods which rely heavily on temporal information. The Iterated Closest Point method, a greedy method for minimizing the distance between model and depth map, is commonly used [29, 33], and [34], as the basic framework for tracking skeletal pose. These tracking approaches often additionally require an accurate skeletal model beforehand. Therefore, in [1] and [32], methods for simultaneous human shape estimation and tracking were also introduced. The simplicity of greedy algorithms in generative models has computational advantage over the discriminative models. The recent tracking algorithm of [3] can operate in 125 fps with uniform sub-sampling. Furthermore, the temporal information can be a powerful cue in pose estimation. For the available public pose DBs, tracking methods such as those of [3] and [32] outperform discriminative methods in both computational time and accuracy.

#### Pose Estimation from Single Depth Image

In contrast to tracking methods based on generative models, a discriminative approach aims to directly train the conditional probability of the body part labels or joint positions. This enables pose estimation even from a single depth image, without any initialization from the previous frame or accurate body models [5, 6, 10, 35]. In [31], Ye *et al*. initialized the skeletal frame by alignment and database look up, and the final pose was refined by minimizing least-squares distance. Similarly, in [4], Baak *et al*. estimated the pose from a single frame by nearest neighbour learning on extrema points. The methods based on randomized decision and regression forests have shown to be effective and efficient [5, 6, 10, 36, 37], where one such method is able to operate in real-time and on commercial products [37]. In the work of Shotton *et al*. [5], decision forest is traversed to find the body part labels for each pixel. Once the pixels are classified into body parts, the possible joint positions are found with multiple mean-shifts. The pixel-wise decision forest method was furthered extended by using ideas from Hough forest [36]. In the methods of [10] and [6], the positions of joints are directly estimated from the regression forests by learning the joint offsets from the pixel positions. Parallel implementations of these methods have achieved super-real-time performance [5, 10]. Overall, discriminative methods have an advantage over the generative tracking methods which require a fairly comprehensive human model along with accurate pose estimation in the previous frame. However, compared to the state-of-art body tracking [3], methods using randomized decision trees for pixel-wise inference often require heavier computation with a need for powerful GPU or multi-core processors [5, 10]. Moreover, changes in configuration to improve generalizability and accuracy, such as increasing the number of trees, might induce an additional computational burden.

#### Estimation Methodologies

Methods based on decision forest have been proposed not only for human pose estimation, as mentioned above, but also for face alignment and hand pose estimation. In [38], Cootes et al. applied random forests for regression of face landmark positions. Here, similar to the methods of [5] and [10], random forests are evaluated on uniform grid coordinates which are aggregated by voting to achieve efficient face alignment.

The computation time is improved by sequentially jumping on to offset points for localizing the land-marks in MR brain image registration [14]. Similarly, the number of samples in which regression is performed is greatly reduced in the proposed method by using random walks. While it has also been applied to determine segmentation labels for all pixels [39], random walks is essentially Markov Chain Monte Carlo (MCMC) sampling intended to improve sampling efficiency and has been applied to object tracking [40]. Improving sampling efficiency is a key aspect, also, for the gradient descent method and its variants used for optimization. In [41], the directional vectors of variable increments for optimization are learned, just as the direction for relevant samples are learned by random forests in the proposed method.

Another approach to enhance sampling efficiency is to incorporate the structure of the object within the regression framework. In the method by Tang et al. [42], the learned object structure is used to construct a hierarchical random forest, called latent regression forest (LRF). While the sampling positions for each joint are efficiently selected when training the LRF, the hierarchical tree results in a great number of leaf nodes, requiring an extremely large training data set. In contrast, the proposed method requires much less data since a separate regression tree is trained for each joint, while enabling efficient sampling.

## Materials and Methods

### Forest Walks and Forest Jumps

In this section, the proposed methods are presented, where details of constructing the training set, training the regression trees and the pose estimation algorithm are described. This paper follows the problem addressed by Shotton [5] and Girshick [10], that is, the pose estimation from a single depth image using randomized decision trees for discriminative training. As with these works, the random forests use simple depth comparison features which can be computed extremely efficiently, and are invariant to 3D translation. However, our approach is different from these works in that a regression forest is trained for each human joint in the skeleton, while a regression forest is trained for all body parts in previous methods.

**Fig 2:**
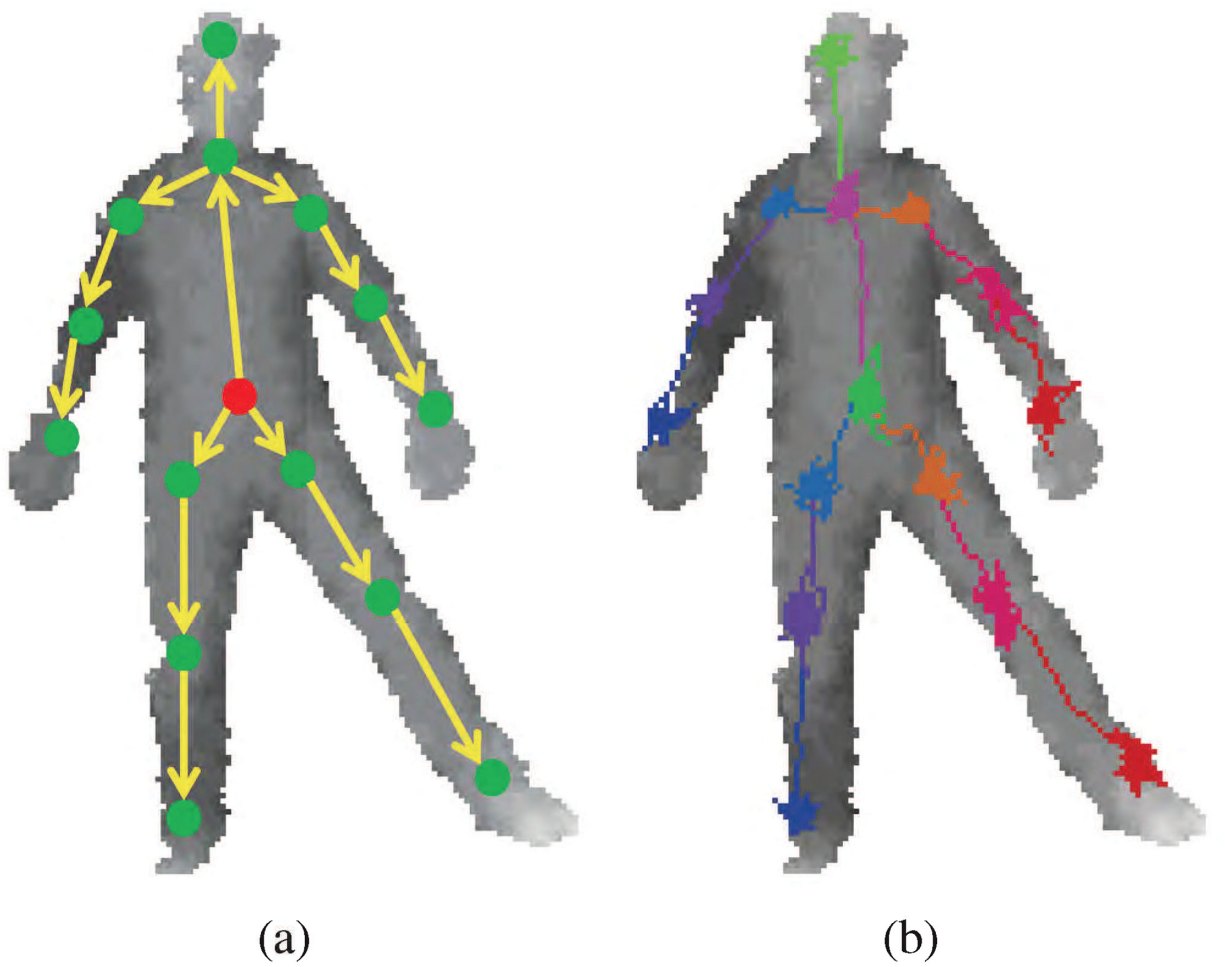
The adjacent joint positions can be used as the starting positions for new RTW. (a) illustrates the kinematic tree implemented along with RTW. First, the random walk toward belly positions starts from body center. The belly positions (red dot in (a)) become starting point for hips and chest, and so forth. (b) shows the RTW path examples.

For localizing problems, a regression tree can be trained for relative offset to the target joint position as in [10] and [36]. Otherwise, a correct position can be found if the direction toward the position is known in any point on the body. Ideally, the directions or offsets to all parts should be trained from all possible positions in the whole body. This will ensure the correct joint position to be found even when starting from a random point as shown in Fig 1. However, this is difficult to train and heavily redundant. Rather, a kinematic tree can be used to reduce the size of regions required for training and to provide a nearby starting point for an adjacent joint such that all position samples are approximately in-line with the skeletal frame. See Fig 2. Details of the method are provided in the following subsections, starting with training sample collection.

#### Training Point Collection

The input data is a single depth image *I* comprising scalar depth measurements of a single segmented human body. For a set of body depth images {*I*_1_, *I*_2_,…}, there are corresponding ground truth skeletal joint positions {*P*_1_, *P*_2_,…}. Each ground truth skeletal pose is represented as 15 body joint positions *P* = (*p*_1_, *p*_2_,…, *p*_15_) with each $p_{j}=(\tilde{x},\tilde{y},\tilde{z})$, *j* = 1,…,15 representing the 3D coordinate of the *j* ^*th*^ joint. The 15 joints in skeletal frame are as follows: head, chest, belly, L/R hip, L/R shoulders, L/R elbows, L/R wrists, L/R knees and L/R ankles. The placement of the each joint is illustrated in Fig 2a.

We train a regression tree that represents the direction or offset toward a particular joint *p*_*j*_ from nearby random point. We thus collect samples of random points from training images with ground truth joints. For each depth *I*_*i*_ and *p*_*j*_ ∈ *P*_*i*_, offset points $q=(\tilde{x}+x_{o},\tilde{y}+y_{o},\tilde{z}+z_{o})$ are sampled with offsets *x*_*o*_, *y*_*o*_, and *z*_*o*_ in each axis having uniform distribution between [−*dist*_*max*_, *dist*_*max*_]. We implement rejection sampling technique to constrain the distance between *q* and *p*_*j*_. The offset sample points are collected from the target joint and adjacent joints based on the expected pathway. For example in Fig 2, the starting point for finding head position is from chest. Therefore, the offset sample points are collected from head and chest joint positions.

The offset is *u* = (*p*_*j*_ − *q*). The unit direction vector to the joint from an offset point is found by $\hat{u}=(p_{j}-q)/\|p_{j}-q\|$. A training sample *S* for direction regression tree consists of body depth image *I*, random offset point *q*, and the unit direction vector $\hat{u}$ toward the true joint position.

$$\begin{array}{c} S=(I,q,\hat{u}). \end{array}$$ (1)

A training sample for offset regression tree has *u* instead of $\hat{u}$.

$$\begin{array}{c} S=(I,q,u). \end{array}$$ (2)

**Fig 3:**
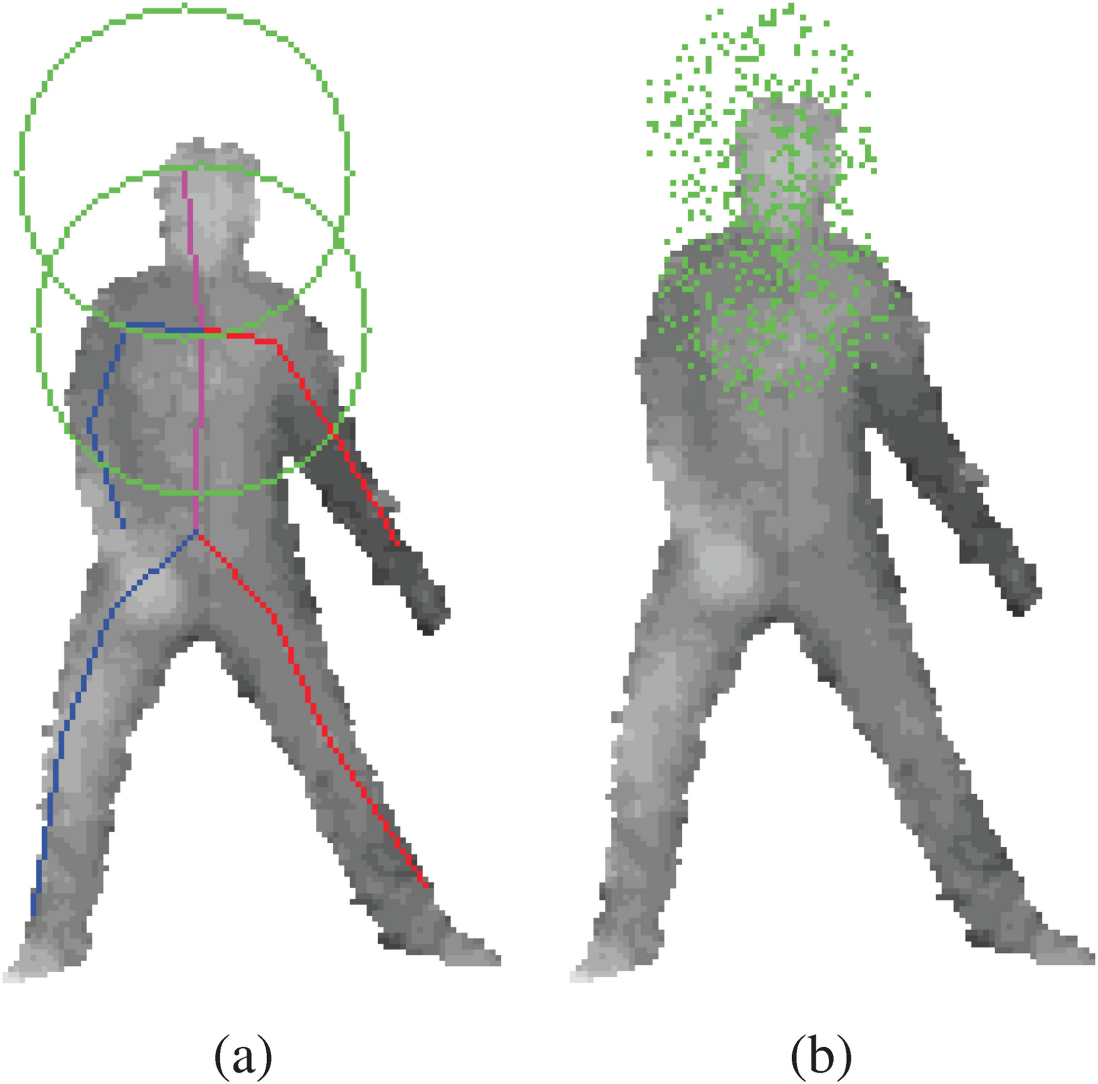
The offset positions are randomly sampled from head and chest joints. (a) illustrates offset sample range spheres in green. In (b), the green dots represents offset samples.

Compared to the previous random forest pose estimation methods, our approach trains a separate regression for each body joint, and the objective function is a simple sum of 3D squared errors. In Girshick et. al’s paper [10], a single regression tree is trained to learn offsets to all body parts. This leads to very large memory requirement at the leaves, and a very high dimensioned error sum to be minimized. They employ vote compression at the leaves. Also, per joint distance thresholds are used to eliminate joint offsets that are too far away. In our approach, each tree is specialized for each body joint, which allows for different and appropriate training set for each joint. For example, in order to train for head, we only need samples around the head as exemplified in Fig 3. Points around feet are not included in the training set for the head because features around feet will not likely tell you where the head is. Like this, we are able to naturally eliminate irrelevant samples from the training set. In the previous approach, all offsets to body joints are trained by a single tree, which is not an effective and efficient approach [10] without the additional per joint distance thresholds and vote compressions. We were able to circumvent the two major problems addressed by [10], by finding a way to train specific regression tree for each joint.

#### Training Regression Tree

During training of the regression tree, the training samples are separated into different partitions using depth image feature and the offset position (*I*, *q*). The goal is to find partitioning binary tree that minimizes the sum of squared difference of $\hat{u}$. The objective function is defined as follows.

$$\begin{array}{c} E^{reg}\left(Q\right)=\sum_{Q_{s}\subset Q}\sum_{\hat{u}\in Q_{s}}{∥\hat{u}-{\bar{u}}_{s}∥}^{2}, \end{array}$$ (3)

$$\begin{array}{c} {\bar{u}}_{s}=\frac{1}{|Q_{s}|}\sum_{\hat{u}\in Q_{s}}\hat{u}, \end{array}$$ (4)

where **Q** is a set of partition *Q*_*s*_ of training samples. ${\bar{u}}_{s}$ is the average of unit *direction* in the training sample partition *Q*_*s*_. The Eqs (3) and (4) are the objective function for training unit directions. For training *offset*, $\hat{u}$ can be replaced with *u*. This is also true for following Eq (5), which explains the split criteria for each tree node.

The training samples are recursively partitioned into the left and right child nodes denoted as *Q*_*l*_(*ϕ*) and *Q*_*r*_(*ϕ*), respectively. Here, *Q*_*l*_(*ϕ*) ∪ *Q*_*r*_(*ϕ*) is the training sample set at the parent node and *Q*_*l*_(*ϕ*) ∩ *Q*_*r*_(*ϕ*) = ∅. At each parent node, a split parameter *ϕ* is randomly selected to minimize the variance at left and right partitions.

$$\begin{array}{c} \phi^{*}={argmin}_{\phi }\sum_{\hat{u}\in Q_{l}\left(\phi \right)}{∥\hat{u}-\bar{u}∥}^{2}+\sum_{\hat{u}\in Q_{r}\left(\phi \right)}{∥\hat{u}-\bar{u}∥}^{2}, \end{array}$$ (5)

$$\begin{array}{c} where\;\left|Q_{l}\left(\phi \right)\right|>N_{min},and\left|Q_{r}\left(\phi \right)\right|>N_{min}, \end{array}$$ (6)

where *N*_*min*_ minimum cardinality constraint on the sizes of child nodes. If the minimum size criteria cannot be met, no further split is considered.

The feature used for partition is defined, similar to that in [5], as:

$$\begin{array}{c} f_{\theta }\left(I,x\right)=d_{I}(x+\frac{t_{1}}{d_{I}\left(x\right)})-d_{I}(x+\frac{t_{2}}{d_{I}\left(x\right)}), \end{array}$$ (7)

where parameters *θ* = (*t*_1_, *t*_2_) are offsets to the current pixel coordinate position *x*. *d*_*I*_(*x*) is the depth at *x*. The division by the depth *d*_*I*_(*x*) acts as transformation from world space coordinate to pixel coordinate. As with previous random forest approaches [5], *d*_*I*_(*x*) outside of human segmentation is given a large positive constant value.

Given *θ* together with threshold *τ*, *ϕ* = (*θ*, *τ*) partitions samples in parent node into left and right subsets *Q*_*l*_ and *Q*_*r*_ as:

$$Q_{l}\left(\phi \right)=\{S_{i}\;|\;f_{\theta }\left(I,x\right)<\tau \}.$$ (8)

$$Q_{r}\left(\phi \right)=Q\;\backslash \;Q_{l}\left(\phi \right).$$ (9)

The split parameters *ϕ* are randomly chosen from uniform distribution. For each selection of *ϕ*, the partitions *Q*_*l*_(*ϕ*) and *Q*_*r*_(*ϕ*) are evaluated with Eq (5), and the best ϕ* is saved as the final split parameters of the node. The split parameters are found down the tree until the minimum sample number criteria Eq (6) cannot be met, else until the maximum number of leaf size is reached. In this paper, the maximum leaf size is fixed at 32768. The leaf size of 32768 is equivalent to a full binary tree of maximum depth 15.

#### Leaf Nodes

When regression tree is trained, each training sample will correspond to one of the leaf bins. A leaf node in a regression tree represents a set of training samples *Q*_*s*_ = {*S*_1_, *S*_2_, *S*_3_,…}. For leaf node for greedy algorithms like GFW and GFJ, the leaf node simply contains the average of training samples that reached the leaf node, $\bar{u}$.

$$L_{t}=\left\{\bar{u}\right\}.$$ (10)

For training the offset, the average of *offset* is stored at the end of leaf node. The average of *direction* is normalized $\hat{u}=\bar{u}/||\bar{u}||$ when training for the unit direction. ${ℒ}_{t}=\{\hat{u}\}.$

For RFW and RFJ algorithms, we want to add randomness in the selection of new sample position in order to provide a way to escape local minima. In the same spirit as Markov Chain Monte Carlo (MCMC) optimization approaches [43], we rely on the stochastic relaxation in choosing the unit direction or offset. The training samples at the leaf nodes *Q*_*s*_ are further clustered using *K*-means algorithm. The goal is to construct representative vectors ${\bar{u}}_{k}$ that minimize the variance *V* of unit direction vectors $\hat{u}$ that is defined by

$$V=\overset{K}{\underset{k=1}{\sum }}\underset{\hat{u}\in C_{k}}{\sum }∥\hat{u}-{\bar{u}}_{k}{∥}^{2},$$ (11)

$${\bar{u}}_{k}=\frac{1}{\left|C_{k}\right|}\underset{\hat{u}\in C_{k}}{\sum }\hat{u},$$ (12)

where *C*_*k*_ is the *k* ^*th*^ cluster of *Q*_*s*_. The clusters are found using typical *K*-means algorithm, using random initialization. Then, the average unit directions ${\bar{u}}_{k}$ are normalized as ${\hat{u}}_{k}={\bar{u}}_{k}/\|{\bar{u}}_{k}\|$. Traversing to the end of tree will produce a set of unit direction vectors and proportional size of clusters. Leaf set ℒ is defined as follows:

$$\begin{array}{c} {ℒ}_{t}=\left\{\left(\frac{\left|C_{1}\right|}{\left|Q_{s}\right|},{\hat{u}}_{1}\right),\ldots ,\left(\frac{\left|C_{K}\right|}{\left|Q_{s}\right|},{\hat{u}}_{K}\right),\right\}. \end{array}$$ (13)

The direction of the step is chosen randomly from one of the unit vectors ${\hat{u}}_{k}$ in Eq (13). Again for offset training, $\hat{u}$ should be replaced with $\bar{u}$.

#### The Regression Forest

A forest is an ensemble of randomized trees. The construction of each tree in the forest is based on the bagging technique where the training samples are randomly re-sampled [44]. A training sample includes both depth frame and an offset point. In previous approaches, the depth frames are re-sampled but the offsets or pixel positions were not re-sampled [5, 10]. In this paper, we re-sampled the offset points instead of re-sampling the depth frames. Since the offsets were randomly sampled anyway, this is easier extension to bagging technique. The features and thresholds at each nodes are also re-sampled during training. For each body joint, three trees were trained and combined to make a forest.

In a typical regression forest, the learned distribution from leaf nodes are averaged. In our approach, the leaf results from Eqs (10) and (13) are simply combined into an union.

$$\begin{array}{c} {ℒ}_{F}={ℒ}_{1}\cup {ℒ}_{2}\cup {ℒ}_{3}. \end{array}$$ (14)

The elements of the forest leaf contain all the leaf elements from three trees. In RFW and RFJ, the direction and positions are randomly selected from ℒ_*F*_. The selection probability is still proportional to the weight of the unit direction. For algorithms GFW and GFJ, the next sample position is found by averaging the tree results in ℒ_*F*_.

#### Localization Algorithms

Alg. 1, 2, 3, and 4 summarize respective RFW, RFJ, GFW, and GFJ algorithms for localizing a joint position from series of constant steps and jumps. The algorithms begin with depth map *I*, starting position *q*_0_, regression forest {*T*_1_, *T*_2_, *T*_3_}, and number of steps *N*_*s*_. RFW and GFW have additional step size *dist*_*s*_ parameter. The goal is to find the expectation $\bar{q}$ of sample positions, which provide position for a single joint.

*I* is provided by the depth camera along with human segmentation. {*T*_1_, *T*_2_, *T*_3_} is trained for a specific joint where each tree has randomly produced training samples. *N*_*s*_ and *dist*_*s*_ are adjustable parameters which can provide trade-off between precision and computation time. The efficient starting position *q*_0_ will be varied upon the joint being localized. The leaf nodes are found deterministically, but the direction or offset can be chosen randomly from set of *K*-mean clustered unit vectors at the union of leaves. In GFW and GFJ algorithms, the directions and offsets are simply averaged to find the next position sample.

**Algorithm 1**: The Random Forest Walk (RFW) Algorithm

**Data**: Depth map *I*, starting point *q*_0_, regression forest {*T*_1_, *T*_2_,…}, number of steps *N*_*s*_, and step distance *dist*_*s*_.

**Result**: The average joint position $\bar{q}$.

Initialization. *m* = 0, *q*_*sum*_ = (0,0,0).

**while** *m* < *N*_*s*_ **do**

 Find the leaf node ℒ_*i*_ of *T*_*i*_ using (*I*, *q*_*m*_).

 Set of tree leaf is found ℒ_*F*_.

 Randomly select ${\hat{u}}_{k}$ from ℒ_*F*_ with probability

$Prob(k)\propto \frac{|C_{k}|}{|Q_{s}|}$.

 Step into new position using *k* ^*th*^ direction vector.

$q_{m+1}=q_{m}+{\hat{u}}_{k}\cdot dist_{s}$.

 Update joint position sum *q*_*sum*_ += *q*_*m*+1_.

 Update *m* += 1.

**end**

Compute the joint position by averaging step positions. $\bar{q}=q_{sum}/N_{s}$.

**Algorithm 2**: The Random Forest Jump (RFJ) Algorithm

**Data**: Depth map *I*, starting point *q*_0_, regression forest {*T*_1_, *T*_2_,…}, and number of steps *N*_*s*_.

**Result**: The average joint position $\bar{q}$.

Initialization. *m* = 0, *q*_*sum*_ = (0,0,0).

**while** *m* < *N*_*s*_ **do**

 Find the leaf node ℒ_*i*_ of *T*_*i*_ using (*I*, *q*_*m*_).

 Set of tree leaf is found ℒ_*F*_.

 Randomly select ${\bar{u}}_{k}$ from ℒ_*F*_ with probability

$Prob(k)\propto \frac{|C_{k}|}{|Q_{s}|}$.

 Step into new position using *k* ^*th*^ offset.

$q_{m+1}=q_{m}+{\bar{u}}_{k}$.

 Update joint position sum *q*_*sum*_ += *q*_*m*+1_.

 Update *m* += 1.

**end**

Compute the joint position by averaging step positions. $\bar{q}=q_{sum}/N_{s}$.

#### Kinematic Tree Pose Estimation

Since proposed localization algorithms can operate independently for each joint, all joint positions can be found in parallel. However, a sequential estimation will provide better starting point *q*_0_. A starting point closer to the targeted joint position is desired because the forest walk will reach the joint position faster. With known skeletal topology, the firstly estimated joint position can provide the starting point for next adjacent joint.

The joint positions are determined sequentially according to the typical skeletal topology. This joint estimation sequence is illustrated in Fig 2. First, the belly position is found by starting from the body center which is an average position of depth pixels. Then, the expectation $\bar{p}$ of belly position is used as the starting point *q*_0_ of next adjacent joint. An example of the sequential RFW is shown in Fig 2b. Note that the computation for each limb may be processed in parallel, if further computational time reduction is desired.

**Algorithm 3**: The Greedy Forest Walk (GFW) Algorithm

**Data**: Depth map *I*, starting point *q*_0_, regression forest {*T*_1_, *T*_2_,…}, number of steps *N*_*s*_, and step distance *dist*_*s*_.

**Result**: The average joint position $\bar{q}$.

Initialization. *m* = 0, *q*_*sum*_ = (0,0,0). **while** *m* < *N*_*s*_ **do**

 Find the leaf node ℒ_*i*_ of *T*_*i*_ using (*I*, *q*_*m*_).

 Set of tree leaf is found ℒ_*F*_.

 Find average of unit directions $\bar{u}=avg({ℒ}_{F})$.

 Normalize into unit direction $\hat{u}=\bar{u}/\left\|\bar{u}\right\|$.

 Step into new position using $\hat{u}$ direction vector.

$q_{m+1}=q_{m}+{\hat{u}}_{k}\cdot dist_{s}$.

 Update joint position sum *q*_*sum*_ += *q*_*m*+1_.

 Update *m* += 1.

**end**

Compute the joint position by averaging step positions. $\bar{q}=q_{sum}/N_{s}$.

**Algorithm 4**: The Greedy Forest Jump (GFJ) Algorithm

**Data**: Depth map *I*, starting point *q*_0_, regression forest {*T*_1_, *T*_2_,…}, and number of steps *N*_*s*_.

**Result**: The average joint position $\bar{q}$.

Initialization. *m* = 0, *q*_*sum*_ = (0,0,0). **while** *m* < *N*_*s*_ **do**

 Find the leaf node ℒ_*i*_ of *T*_*i*_ using (*I*, *q*_*m*_).

 Set of tree leaf is found ℒ_*F*_.

 Find average of unit directions $\bar{u}=avg({ℒ}_{F})$.

 Step into new position using $\bar{u}$ offset.

$q_{m+1}=q_{m}+{\bar{u}}_{k}$.

 Update joint position sum *q*_*sum*_ += *q*_*m*+1_.

 Update *m* += 1.

**end**

Compute the joint position by averaging step positions. $\bar{q}=q_{sum}/N_{s}$.

## Experimental Evaluation

We validate our algorithms on two public 3D pose datasets, namely, the SMMC-10 set http://ai.stanford.edu/~varung/cvpr10/ [2] and the EVAL set http://ai.stanford.edu/~varung/eccv12/ [3]. The SMMC-10 set comprises 28 action sequences performed by a single individual. The more recent EVAL set comprises 3 models performing 8 sequences each. It is more straightforward to train and test the proposed method using the EVAL set since it provides multiple models for train and test set separation. Specifically, we train the proposed methods using the sequences of two models and test on the sequences of the remaining model. This process is repeated three times so that sequences for each model is tested at least once, in a typical leave-one-out training scheme. For testing on the SMMC-10 set, we constructed our own training set with manually labelled skeleton positions since it contains only a single individual. Due to the completeness of training and testing on the dataset, the majority of evaluation results are obtained from the EVAL set.

The mean average precision is evaluated against the recent state-of-the-art pose estimation algorithms. The step size, the required step numbers, and the number of trees in the forest were adjusted in the evaluation. In our experiments, a sufficient accuracy and stability is achieved with only 64 steps. By fixing the number of steps, the computation time is stable regardless of the depth image size or occlusions in joints. With 15 joints overall in the skeletal model, the total number of tree traverses is just under one thousand.

A conventional precision measure for pose estimation algorithms is the 10 cm rule [2, 5, 10]. If the estimated joint position is within 10 cm of the ground truth, it is considered correctly estimated. The precision of the estimated twelve joints on each frame are averaged together to find mean average precision (mAP). The joints included in the evaluations are head, chest, L/R shoulders, L/R elbows, L/R wrists, L/R knees, and L/R ankles. The evaluation joints are chosen based on the availability of ground truth.

The properties of forest walk sub-sampling methods are evaluated in terms of step size and step number. From experiments over various step sizes, we choose best performing step sizes for RFW and GFW algorithms. RFW, GFW, RFJ, and GFJ algorithms are compared with each other in regards to step/jump and tree number. The trade-off between computation time and precision can be derived from the number of position samples and the number of trees in a forest. The computation and precision of the proposed method is compared with previous methods based on both tracking and discriminative approaches [3, 5, 10, 32].

### Step Sizes for Walking

Intuitively, larger number of random or greedy walk steps will result in more accurate or otherwise more stable pose estimation. Since the directions of steps are chosen from randomized forests, there is always a chance for a wrong direction. A large number of steps will hopefully average out the errors. At the same time, unnecessary computation time should be avoided. If a further increase in the number of steps does not result in significant increase of precision, the saturation number of steps has been reached.

**Fig 4:**
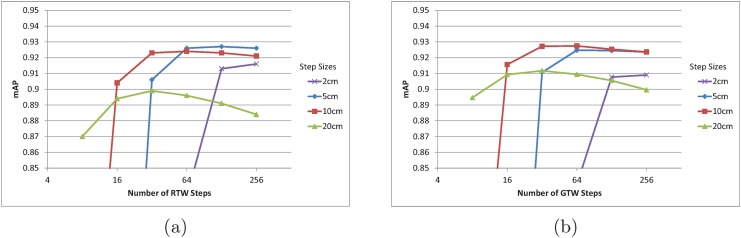
(a) mAP of RTW with varying step numbers and sizes for the EVAL sequence. (b) mAP of GTW with varying step numbers and sizes for the EVAL sequence. In (a) and (b), each line represents different step sizes.

Different step sizes have different saturation numbers. If each step is large, the targeted joint position will be reached quickly, provided that the direction selections are mostly correct. If the step size is too large, however, the steps may keep jumping over the correct joint position, and it might step into a new position where it cannot find correct direction toward the target joint. For random and greedy walk using a single tree, the step sizes were varied among 2 cm, 5 cm, 10 cm, and 20 cm in the evaluation as presented in Fig 4. The mAP for different step size and number of steps are tested for the EVAL set. The number of steps are varied from 8, 16, 32, 64, 128, and 256.

Overall, the precision starts to saturate early for larger step sizes. The saturation number for the step sizes of 2 cm, 5 cm, 10 cm and 20 cm are 128, 64, 32, and 16 respectively for random walk. Overall, the greedy walk algorithm has slightly lower precision saturation number, because greedy walk always moves toward the target joint unlike the random walk algorithm. However, the greedy walk algorithm’s precision drops more as the step number increases awhile random walk algorithm drops less. When walking with small step sizes, the randomness of the direction is able to average out wrong step directions once it reached the target joint position.

In Fig 4, the highest precision 0.927 is achieved for both random and greedy walk with 5 cm and 10 cm step sizes, respectively. Both methods achieve over 1000 fps and the mAP is the state-of-the-art. The mAP can be further improved with increasing number of trees in forest. For the experiments with multiple trees, we will fix the step sizes for RFW and GFW to 5 cm and 10 cm, respectively.

### Comparison between Proposed Methods

**Fig 5:**
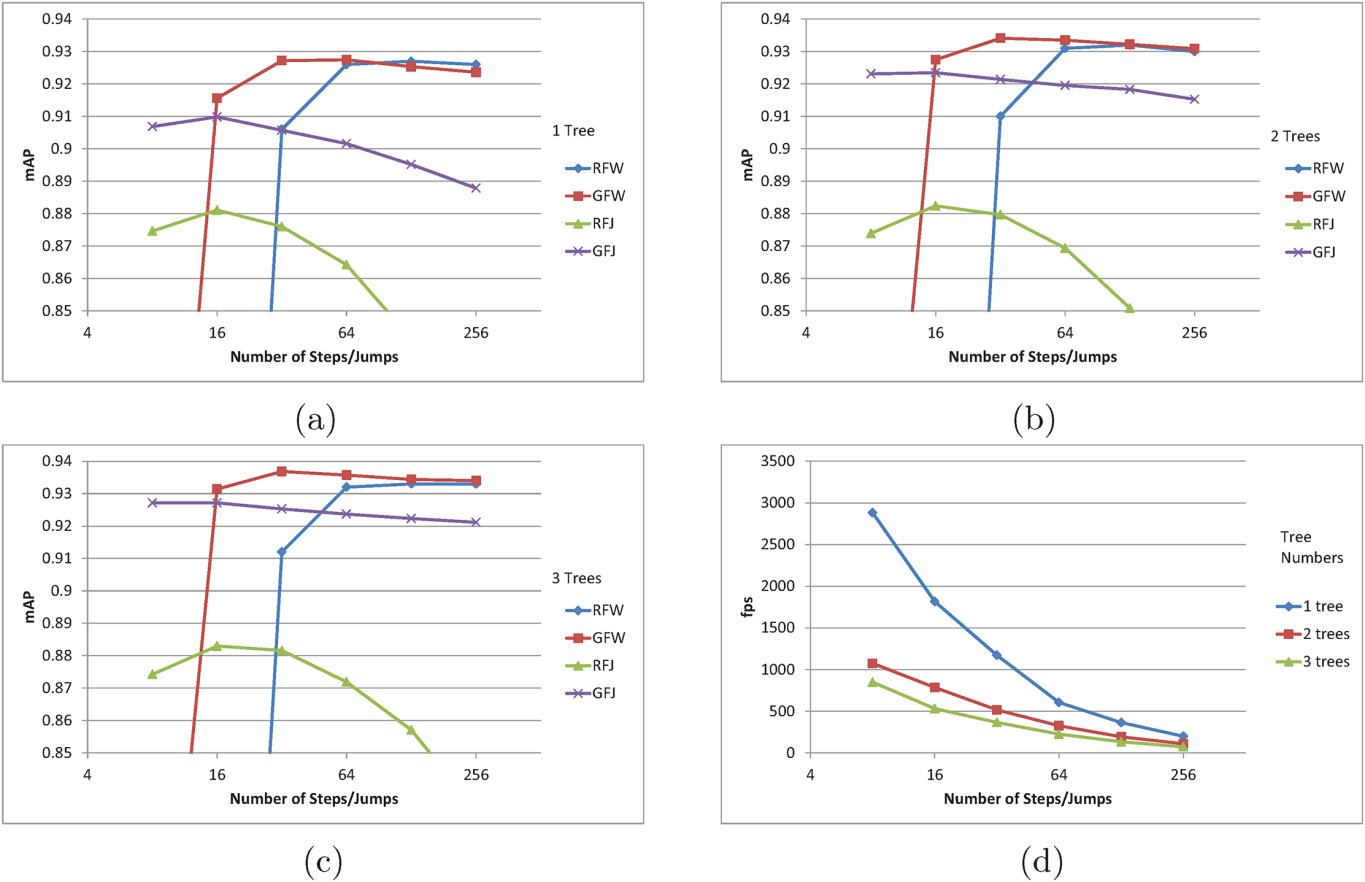
(a) mAP using 1 tree. (b) mAP using 2 tree forest. (c) mAP using 3 tree forest. (d) Average fps. In (a) (b) and (c), mAPs of proposed pose estimation algorithms are shown in respect to the number of steps and jumps taken. An additional accuracy gain can be observed for an increased tree number in random forest. (d) shows the average fps for different number of samples and trees.

The proposed RFW, GFW, RFJ, and GFJ methods are compared across different tree numbers in the forest. See Fig 5. For all four methods, the inclusion of additional trees in the forest increased the mAP as expected. The highest precisions are consistently obtained by GFW algorithm. RFW algorithm achieved the similar mAP to that of GFW when using only 1 tree. However, when the number of trees is increased in the forest, GFW algorithm has notably higher mAP than RFW as shown in Fig 5c. As the number of trees increases, the accuracy of random forest output also increases, and thus it becomes more sensible to trust the average direction than to randomly select direction from the distribution. Otherwise, mAP of RFW and GFW converge to the same mAP as the number of steps become higher.

The forest jump methods’ mAP have opposite characteristic to forest walk algorithms’ mAP in terms of the jump numbers. Forest jump methods’ highest precisions are achieved at 8 jumps, and the precisions are lowered as the jump number increases. Unlike the forest walk methods, the forest jump methods allow for large change in the sampling position in as single traverse of the random forest. This makes it more likely for forest jump methods to jump out of the correct joint position and get stuck in an area, unable to jump back to the correct position. We can also see how large step size results in low mAP for the forest walk methods in Fig 4. RFJ method’s mAP shows the most dramatic decrease.

The number of steps/jumps are plotted against the fps in Fig 5d. The fps was computed by averaging the computation time over all four methods. At 8 position samples per joint, the proposed algorithm can output faster than 2500 fps with 0.907 mAP which is higher than the recent implementation by Ganapathi et al. [3]. RFW of eight jumps are taken each for 15 joints, translating into 120 (8 × 15) traversals of a single level 15 tree. The highest mAP is achieved by GFJ algorithm with 0.937 mAP at 365 fps which is faster and more accurate than any other preceding methods. In Table 1, we list few numerical results with different fps and mAP.

**Table 1:** Few notable results from Fig 5 are shown in comparison to recent method by Ye et. al [32].

Algorithm	GFJ	RFW	GFW	Ye et. al
Tree Number	1	1	3	N/A
Steps/Jumps	8	128	32	N/A
mAP	0.907	0.927	0.937	0.921
fps	2680	1262	325	+30(GPU)

The size of steps can be viewed as a parameter that controls the damping in control theory. In the evaluation, we saw that the largest step size tends to miss the correct joint position by overshooting, and the smallest step size tends to take more time in reaching the correct position, which may be viewed as an overdamped system. For forest jump approach, which trains for the correct offsets, we might expect a critically damped system, however in the evaluation, we saw that forest jump approach has smaller accuracy than the forest walk algorithms. The accuracy difference may be due to several reasons. First, the trained step-size or the offset size of forest jump algorithm is not effective as the manually tuned step-size of forest walk algorithms. Second, training for the offset using the regression forest may not be as effective as training for the directions, which has one less dimension due to the uniform length.

When comparing the random or greedy approaches, it is interesting to see Fig 4. When the step size is the smallest, the random walk approach has higher mAP than greedy walk. When the step size is the largest, the accuracy of greedy walk approach becomes higher. This can be explained in terms of local maxima dilemma in optimization problems. In order to gain high fps, the proposed approaches sample only a small number of positions which corresponds to local regions in the 3D space. If the step size is small, the local sampling region becomes small, however employing the randomness to the steps allows for a chance to escape from local maxima to sample from the global maxima regions. When the step size is large, the sampling region is already sufficiently large, and the randomness only hinders to pinpoint the global maxima. As with simulated annealing, the temperature or the parameter that controls the randomness of a process has different optimal value for each system.

### Comparison with Existing Methods

**Fig 6:**
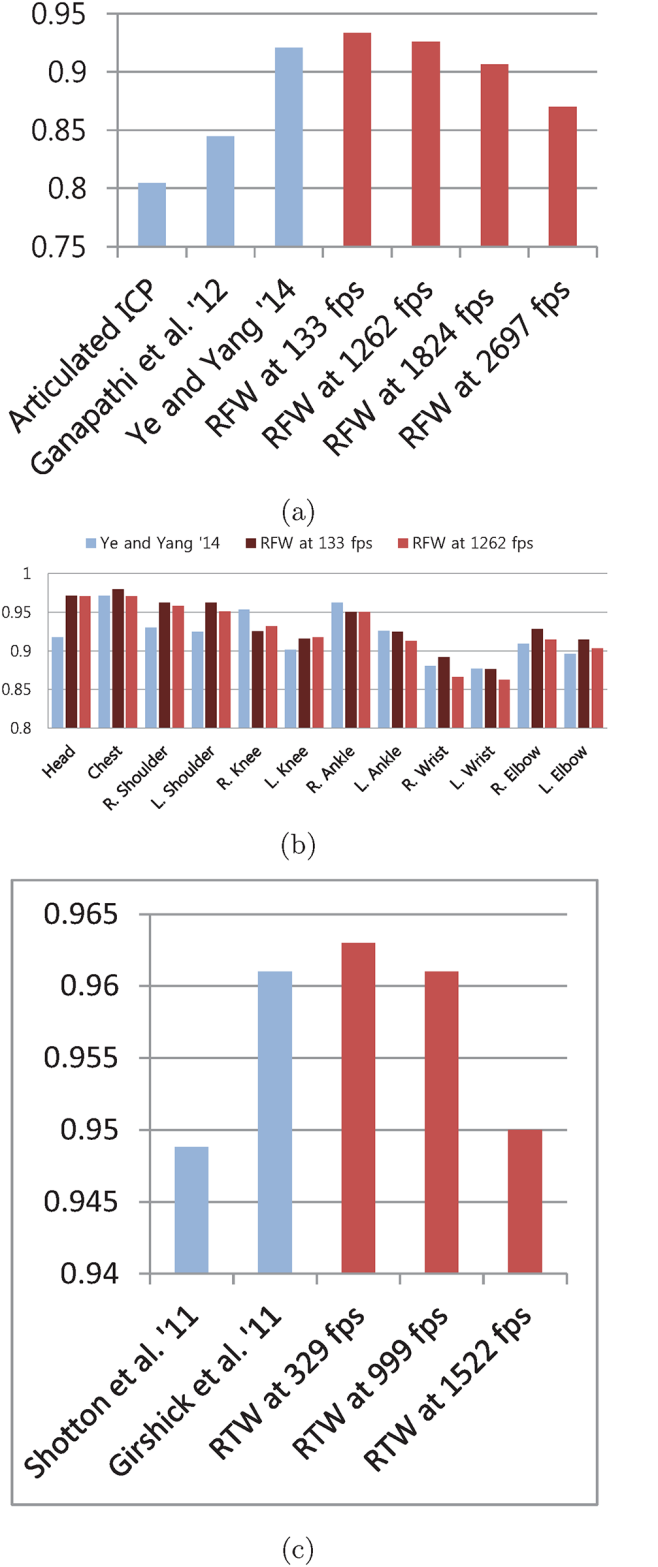
(a) mAP for EVAL set. (b) mAP of each joint for EVAL set. (c) mAP on SMMC-10 set. The proposed approach is compared with the recent algorithms using EVAL [3] set in (a) and (b). (a) shows our 3 results in red with different fps. RTW approach performs slightly higher than Ye and Yang [32] at 1262 fps. Even at 2687 fps, RTW shows higher precision than Ganapathi et al. [3]. The precisions in each joint are presented in (b). (c) compares results for SMMC-10 test sequence. Shotton et al. [5], Girshick et al. [10] and our approach are methods for pose estimation from a single image.

The recent state-of-the-art pose tracking algorithm implementation by Ye and Yang [32] showed 0.921 mAP on EVAL sequences [32]. Their implementation achieved faster than 30 fps operation using GPU. The pose tracking method by Ganapathi et al. [3] does not rely on GPU to achieve 125 fps operation, but the precision is significantly lower. In Fig 6a, the proposed GFW is compared with these two methods [3, 32]. Our method is able to obtain slightly higher precision than Ye and Yang’s state-of-the-art tracking algorithm [32]. In addition, the computation is more than 40 times faster than their GPU assisted implementation [32].

Another advantage of our approach over previous tracking-based algorithms comes from the ability to estimate pose from a single depth image. In our approach, the pose of previous frame is not used as either the initial pose or a-priori knowledge for the estimation of pose in current frame. In that regard, the proper comparison should be made with the single depth image pose estimation methods of [5, 10]. However, since their evaluation results for the EVAL set are unavailable, the older SMMC-10 test sequences are used in the comparison test. See Fig 6c. Since SMMC-10 set mostly contains relatively easy poses, the results of each method are all fairly high, above 0.94 mAP.

Similar to the EVAL set, the proposed RTW obtains slightly higher precision for the SMMC-10 set, but the computation gain is significant. The previous methods’ 8 core CPU implementations run at 50 and 200 fps [5, 10]. In comparison, the proposed RTW runs at approximately 1000 fps using a single core CPU, awhile obtaining equal or higher mAP.

**Fig 7:**
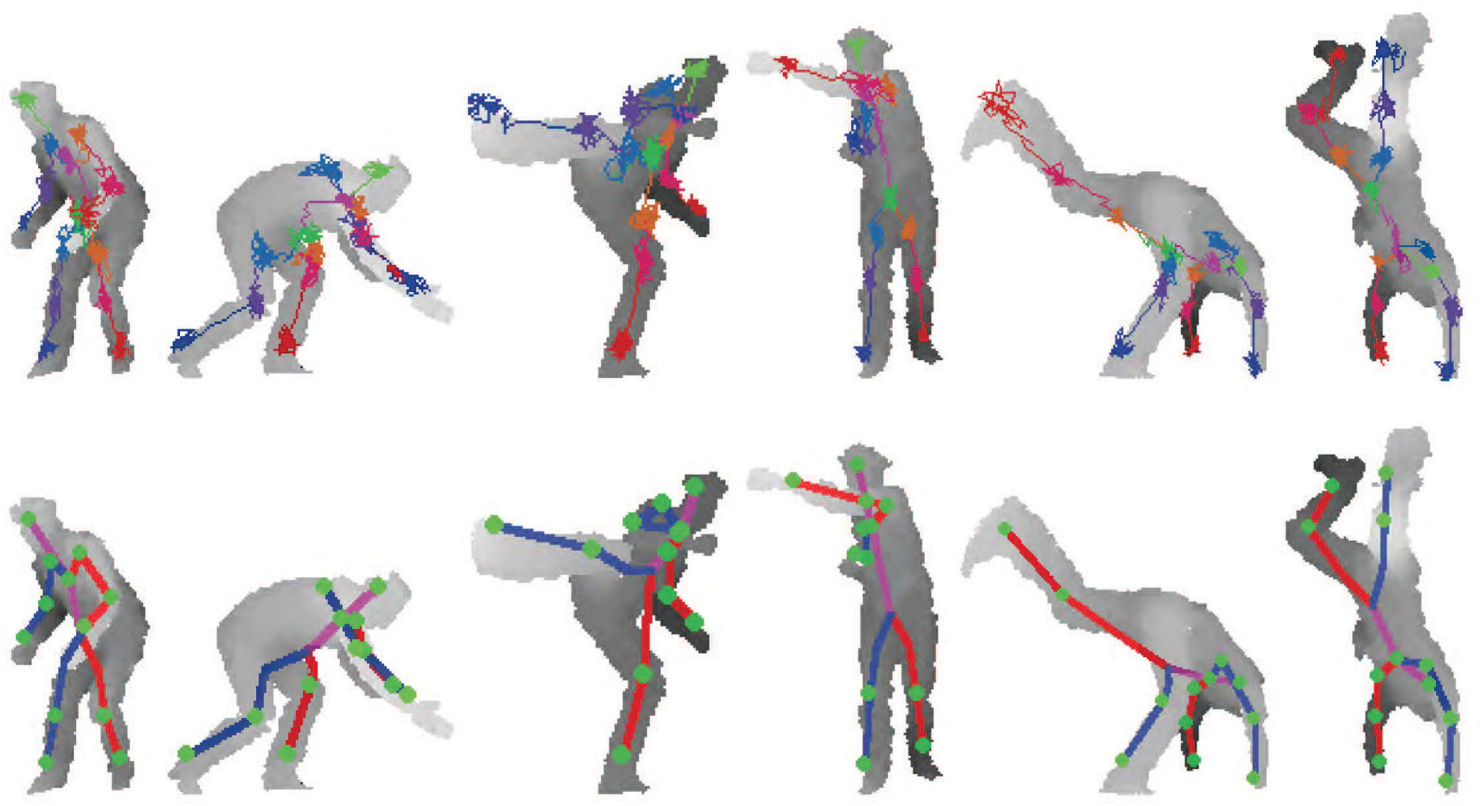
Example results of the RTW from EVAL test data. 64 RTW steps are taken for each joint. The RTW paths are drawn at the top row, the expectations of RTW steps are used to find joint positions in bottom row. The pose estimation from a single image takes less than 1 millisecond.

Finally, few qualitative examples of our method from EVAL test sequences are shown in Fig 7. In the figure, 64 forest walk steps are taken with 5 cm step size. Both the forest walk paths and pose expectations are shown. Note that very hard poses like hand-stand, crouching, and cross-punch are efficiently and accurately estimated with our approach.

## Discussion

In this paper, we proposed several approaches for 3D pose estimation problem, which allow for a large computation gain without decrease in accuracy. The proposed approaches move away from pixel-wise traverses of random forest, and apply a sequential position sampler in the form of random walks and jumps. By initializing the starting point to the adjacent joint according to kinematic tree, we present a practical super-real-time pose estimation algorithm. The obvious computational advantages are demonstrated, and the highest precision are achieved for the public pose DB. Our methods’ ability to trade-off precision and computation time allows for a robust solution to different platforms.

It is worth noting that our work could be effectively applied to various pose estimation problems such as 3D hand poses or gestures estimation problems. Our work could be extended to real-time human pose estimation problem for video sequence in future work. Also, it could be applied to outdoor as well as indoor scenes using depth information from stereo matching.
